# Recent Results of Experiments upon the Brains of Animals

**Published:** 1882-02

**Authors:** 


### RECENT RESULTS OF EXPERIMENTS UPON THE BRAINS OF ANIMALS.

The *London Lancet* in its issue of August 20, prints Professors Goltz and Ferrier's accounts of the results of their cutting away the greater parts of the cerebra of animals. It seems that the results respectively varied very greatly indeed to an extent difficult to altogether explain.

Prof. Goltz stated that he commenced experiments six years ago, in which he sought to determine what degree of truth attached to the assertion of Flourens, that large parts of the brain of living animals may be removed without there resulting any apparent loss of cerebral function. He explained his method as consisting of exposing the surface of the brain, and then washing away large portions of its substance by subjecting it to the action of a powerful stream of water. It was found that the statement of Flourens could not be accepted without reservation. After the destruction in the way indicated of large parts of one side of the brain. there results, for instance, hemiplegia, which is, however, not permanent, but transient, with cross blindness. After destruction of large parts of both hemispheres by the same method, Goltz found that there was produced a permanent affection of the senses, which were observed to be dulled to an extraordinary degree, without being, however, lost. During the last two years, Prof. Goltz, abandoning his earlier "washing-away" method, has destroyed limited parts of the cerebral surface by means of circular drills with cutting and tearing edges, which, when rapidly rotated, destroy the part of the brain in which they are placed. By removing large parts of the skull cap, and using such instruments, Prof. Goltz has produced either very localized or very extensive lesions, of which he has studied the results; the dog being invariably the animal chosen for experiment. The results differed according as the anterior or posterior halves of the upper surface of the convolutions were destroyed. Without referring in detail to various experiments of Prof. Goltz's, we have to draw special attention to his remarkable assertion that in the dog, after removal by this procedure of the greater part of the grey matter of the hemispheres, of the motor areas, and of the sensory areas, the animal may recover and live for long periods of time, without exhibiting any definite paralysis whatever. Such a dog, according to Prof. Goltz, can see, taste, smell, feel, yet all these senses are dulled. It can move, yet its movements are clumsy. It has none of the paralysis of movement, and none of the losses of special senses, which the doctrines of Ferrier and of Munk would lead us to predict. Though the animal is not stone blind, and can see, hear and feel in a certain manner, yet perception is greatly interfered with and the intellectual functions are weakened. The dog is in a condition of one demented, and many of its instincts are found to be perverted.

Prof. Goltz having sat down, Prof. Ferrier succeeded him. He said that until it is proved that the phenomena observed by Prof. Goltz are dependent upon the grey matter of the hemispheres, it may be assumed that they are phenomena which require, in the case of the dog, merely the integrity of the great basal ganglion. He called the attention of the section to the fact that Prof. Goltz's experiments had been performed entirely on dogs, and he deprecated the drawing of general conclusions from the experiments performed upon one animal species. Without disputing the facts described by Prof. Goltz, he would bring before the section other facts observed by himself in the case of the monkey, and which entirely corroborated his own views in reference to cerebral localization. Prof. Ferrier pointed out that by Prof. Goltz's method a simple and definite lesion could not be established. During the last two years, however, he, in association with Prof. Gerald Yeo, had employed the galvano-cautery for the establishment of perfectly definite lesions, and by using the antiseptic method of treating the wound, had succeeded in eliminating all chances of inflammation and obtaining a perfect recovery from the actual operative procedure in almost every case. He had thus been able to meet the objections which had been advanced to his earlier experiments—to wit, that he studied for too short a time the effects of the lesions produced, and he had been able to observe that definite lesions of localized regions of the surface of the monkey's brain led to definite and persistent paralysis of motion or losses of special senses. Prof. Ferrier then referred in detail to several of his experiments on monkeys, pointing out that in two of the cases where injuries of motor areas had been inflicted not only was there no recovery, but that when, some months afterward, post-mortem examinations were made, there were found descending degenerations affecting the crus cerebri, the pons, and the spinal cord. In a case where a lesion was inflicted upon both occipital lobes, and angular gyri, affecting the supposed centers for vision, there had resulted no paralysis of motion, no loss of smell or hearing or taste, but the animal became stone blind ; its pupils became widely dilated and fixed, and atrophy of the the optic nerve ensued.

The interest attaching to the discussion on localization was greatly enhanced by the fact that whilst Prof. Goltz had brought one of his dogs from Strasburg, Prof. Ferrier was willing to exhibit two monkeys which he had operated upon some months previously, and in one of which he had procured definite motor paralysis and in the other permanent and absolute deafness. On the afternoon of Thursday the section, joined by such visitors as Prof. Charcot and Virchow, adjourned to the Physiological Laboratory of King's College. They had the opportunity of examining the dog in which Prof. Goltz asserted that he had removed the greater part of both hemispheres, including all the supposed motor and sensory areas. That the operative procedures to which the animal had been subjected, had been extensive was quite obvious from an examination of its skull, large gaps in the continuity of the upper and external walls of which were felt. Saving some clumsiness in its movements, this dog exhibited singularly little which would distinguish it from the normal; it appeared possessed of considerable intelligence, and certainly did not suggest to the onlookers that it was a dog demented. In startling contrast to the dog were two monkeys exhibited by Prof. Ferrier. One of them had been operated upon in the middle of January, the left motor area haying been destroyed. There had resulted from the operation right-sided hemiplegia, with conjugate deviation of eyes and of head. Facial paralysis was at first well marked, but ceased after a fortnight. From the first there had been paralysis of the right leg, though the animal was able to lift it up. The arm it had never been able to use. Lately, rigidity of the muscles of the paralyzed limbs had been coming on. The other monkey, as a consequence of paralysis of its auditory centers, was apparently entirely unaffected by loud noises, as by the firing of percussion caps in close proximity to its head.

What conclusions, it will be asked, are to be drawn from these singularly discordant experiments of Profs. Goltz and Ferrier? As a result of their statements and their demonstrations, are we merely to conclude that the brain of the monkey is constituted differently from that of the dog, and that experiments performed upon the brain of one animal throw no light upon the functions of the brain of the other? Until we know the exact conditions of the experiments in the two cases, it will be impossible to analyze the results obtained, which may be explicable rather by the extent or depth of the lesions inflicted in the two cases than by the hypothesis of a different anatomical conformation. Fortunately the two eminent experimenters, whose graphic accounts of their work had been listened to with marked interest by the section, determined upon a line of conduct destined to throw great light upon their experimental procedures. On one of the days following the discussion and demonstration on cerebral localization, Prof. Goltz's dog and Prof. Ferrier's monkey with motor paralysis were deeply anæsthetized, and then killed. The brains were removed, and were handed to a committee composed of Drs. Klein, Langley, Purser, and Schafer, who have been requested to make an elaborate anatomical and histological examination, with a view to localizing the extent of the lesions inflicted by the experimenters. It may be remarked, however, that when the brains which had been removed from the two animals were exhibited in the Physiological Section, it appeared to the onlookers that the lesions in Prof. Ferrier's case exactly corresponded to that which he had predicted; whilst, unquestionably, in Prof. Goltz's experiment the experimenter had failed in removing considerable portions of the gray matter of the convolutions, including parts at least held by Ferrier to be the seat of motor centers.—*Cincinnati Med. News.*

